# Longitudinal social networks impacts on weight and weight-related behaviors assessed using mobile-based ecological momentary assessments: Study Protocols for the SPARC study

**DOI:** 10.1186/s12889-016-3536-5

**Published:** 2016-08-30

**Authors:** Meg Bruening, Punam Ohri-Vachaspati, Alexandra Brewis, Melissa Laska, Michael Todd, Daniel Hruschka, David R. Schaefer, Corrie M. Whisner, Genevieve Dunton

**Affiliations:** 1 School of Nutrition and Health Promotion, Arizona State University, 550 N 5th Street, Phoenix, AZ 85004 USA; 2 School of Human Evolution and Social Change, Arizona State University, Tempe, AZ USA; 3 Division of Epidemiology and Community Health, University of Minnesota, Minneapolis, MN USA; 4 College of Nursing and Health Innovation, Arizona State University, Phoenix, AZ USA; 5 Institute for Health Promotion & Disease Prevention, University of Southern California, Los Angeles, CA USA

**Keywords:** Social network, Friendship, Obesity, Eating behaviors, Dieting, Physical activity, College freshmen, Emerging adults

## Abstract

**Background:**

The transition from the home to college is a phase in which emerging adults shift toward more unhealthy eating and physical activity patterns, higher body mass indices, thus increasing risk of overweight/obesity. Currently, little is understood about how changing friendship networks shape weight gain behaviors. This paper describes the recruitment, data collection, and data analytic protocols for the SPARC (Social impact of Physical Activity and nutRition in College) study, a longitudinal examination of the mechanisms by which friends and friendship networks influence nutrition and physical activity behaviors and weight gain in the transition to college life.

**Methods:**

The SPARC study aims to follow 1450 university freshmen from a large university over an academic year, collecting data on multiple aspects of friends and friendship networks. Integrating multiple types of data related to student lives, ecological momentary assessments (EMAs) are administered via a cell phone application, devilSPARC. EMAs collected in four 1-week periods (a total of 4 EMA waves) are integrated with linked data from web-based surveys and anthropometric measurements conducted at four times points (for a total of eight data collection periods including EMAs, separated by ~1 month). University databases will provide student card data, allowing integration of both time-dated data on food purchasing, use of physical activity venues, and geographical information system (GIS) locations of these activities relative to other students in their social networks.

**Discussion:**

Findings are intended to guide the development of more effective interventions to enhance behaviors among college students that protect against weight gain during college.

## Background

Life as a college freshman is a period of concentrated and immense change, and one in which the risk of weight gain is especially high [1, 2], along with declines in physical activity (PA) levels [3, 4] and worsening of overall diet quality [5, 6]. A major reason is the transition from parental oversight (i.e., living at home) to relative independence, and one in which both social and physical food and exercise environments change. Importantly, these shifts in eating and PA set the stage for lifelong adult behaviors [7, 8]. This makes college, and especially the freshman year, a time of great opportunity to implement obesity prevention interventions. Yet, college freshmen are less frequently studied in obesity research [7].

Friends may play a critical role in development of obesity among young people, as friends can be highly influential on weight-relevant behaviors like eating and PA [9–16]. However, the role of friends is often missing from standard models explaining obesity risk at both the level of the individual [17, 18] and social groups [19, 20]. Particularly, the mechanisms by which friendship networks are integral to patterns of eating and PA are very poorly described. Having a better scientific grasp of the pathways by which friendship networks impact weight-related behaviors and outcomes is crucial for designing effective behavioral and obesity prevention interventions.

**Fig. 1:**
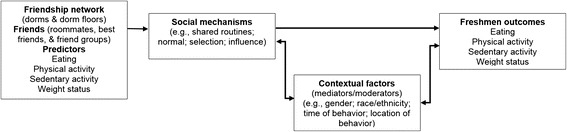
Conceptual framework for the role of friends in weight-related behaviors and outcomes among college freshmen

Strong epidemiological data are needed on the changes (e.g., new friendships, activities/behaviors done together) that occur among friends to better understand the mechanisms impacting friends’ health behaviors/outcomes. The literature presents inconsistent findings about what portion of the relationship between friends’ weight-related behaviors and outcomes can be attributed to different mechanisms such as shared routines, social learning, social pressure, friend selection, friendship ideals, shared access, norms, and influence. This longitudinal study, SPARC (Social impact of Physical Activity and nutRition in College), aims to describe the mechanism(s) by which friends’ and freshmen’s eating/PA behaviors and weight are related and to examine contextual factors related to behaviors among friendship networks over time (see conceptual framework in Fig. 1). Previous studies have examined cross-sectional associations and/or do not measure mechanisms by which behaviors are transmitted among friends; with the exception of studies from the National Longitudinal Study of Adolescent Health, most studies include relatively small and homogeneous samples [21–23]. The current study will gather intensive real-time quantitative data from a large, diverse sample over the course of 1 year with the aim to provide information on behavior and friendship networks, and allow for corroboration of findings through complementary data collection efforts [24]. Using social network analysis methods, we will track friendship selection over the course of a year and assess whether new friends’ behaviors have a greater impact on freshman behaviors compared to longer-term friends. Given the contextual data that are being collected, we will assess how norms and ideals may impact weight-related behaviors and outcomes. Findings will guide the development of effective interventions to enhance behaviors among college students that protect against college weight-gain.

## Methods

### Design overview

The purpose of the SPARC study is to determine mechanisms by which friendship networks impact eating, physical activity and weight among diverse college freshmen. The SPARC study is grounded in a socioecological framework with an emphasis on the interpersonal level by tracking changes in friends’ relationships (perceived, direct report, and social network analysis) with nutrition and PA behaviors, and weight status over a single academic year (9 months). Data will be collected in waves throughout participants’ first year at a large southwestern university from freshmen students living in residence halls. Instruments include web-based surveys, mobile-based ecological momentary assessments (mEMAs), and student card data (time-dated data on food purchasing, use of physical activity venues, and geographical information system (GIS) location of these activities relative to other students in their social networks) from freshmen living in residence halls. The web-based surveys and anthropometrics are scheduled for four time points (the beginning and end of each semester). The m EMAs are scheduled to be administered over four waves. A wave consists of a 1-week period during each of the four target months during the academic year (see Table 1). The student card data are secondary data (times entered dining halls and recreation centers) that will be collected at the conclusion of the study. Each dataset provides unique information and will help us understand the mechanisms and the contextual factors related to friendship networks’ role in students’ eating and PA behaviors, and weight over time (see Table 2 for specific measures).

**Table 1:** Data collection timeline and incentive schedule

Timeline	What	Incentives
August	Web-based survey 1 + anthropometric measurements	$15 Amazon gift cards + swag
September	devilSPARC mobile EMA app (8/day * 4 days)	$5 Amazon gift card for every 10 surveys completed
October	devilSPARC mobile EMA app (8/day * 4 days)	$5 Amazon gift card for every 10 surveys completed
November	Web-based survey 2 + anthropometric measurements	$10 Amazon gift cards + swag
January	Web-based survey 3 + anthropometric measurements	$10 Amazon gift cards + swag
February	devilSPARC mobile EMA app (8/day * 4 days)	$5 Amazon gift card for every 10 surveys completed
March	devilSPARC mobile EMA app (8/day * 4 days)	$5 Amazon gift card for every 10 surveys completed
April	Web-based survey 4 + anthropometric measurements	$10 Amazon gift cards + swag

**Table 2:** Key data collection measures by data collection sources

	Web-based Survey	Ecological Momentary Assessment (EMA)	Student Card
Frequency	4 time points	4 waves (4 days each)	Continuous
Timing	August, November, January, April	October, November, February, March	Continuous
Outcomes (descriptions/examples of measures)			
Dietary intake	During the past month, how often did you: [25] • Eat hot or cold cereals and what kind of cereal • Have any milk and what kind of milk During the past month, how often did you drink: [25, 26] • Regular soda • 100 % pure fruit juice • Coffee or tea with honey or sugar • Sweetened fruit drinks • Sports drinks • Energy drinks During the past month, how often did you eat: [25] • Fruit • Green leafy salad • Fried potatoes • Other kinds of potatoes • Beans (not green beans) • Brown rice or cooked whole grains • Other vegetables • Salsa During the past month, how often did you eat: [25] • Pizza • Tomato sauce • Cheese • Red meat • Processed meat During the past month, how often did you eat: [25] • Whole grain bread • Chocolate or candy • Doughnuts, sweet rolls • Cookies, cakes, pie, brownies • Ice cream, frozen desserts • Popcorn	Are you eating any of the following items? (please check all that apply) [48] • Cookies, sweetened baked goods, candy and frozen desserts • Salty snacks/fried side dishes • Fruits and vegetables (including salads) • Pizza and fast food • Sandwiches (hot and cold), wraps, breads, pitas, and tortillas • Meat, poultry, fish, eggs, and meat alternatives • Pasta, noodles, rice, and other grains • Hot and cold cereals • Other(specify): __________ Are you drinking any of the following items? (please check all that apply) [48] • Sweetened beverages • Coffee or tea drinks • Smoothies • Sports drinks • Energy drinks • Milk • Juice • Water • Other (specify): __________	Location (fast-food, sit-down restaurant, dining hall, convenience store, etc.) Amount of expenditure
Physical activity [30]	In a usual week, how many hours do you spend doing the following activities? [Strenuous exercise/Moderate exercise/Mild exercise] [None, less than 1/2 h a week, 1/2–2 h a week, 2–4 h a week, 4–6 h a week, 6+ h a week]	Select the activity that most closely matches what you are doing (not including responding to this assessment). • Strenuous exercise (heart beats rapidly) (e.g., running, swimming laps, zumba) • Moderate exercise (not exhausting) (e.g., walking quickly, strength training) • Mild Exercise (little effort) (e.g., walking slowly, yoga) In total, how much time will you spend on this activity? [<30 min, 30 min–1 h, 1–2 h, >2 h]	Check-ins at campus exercise facilities
Sedentary activity [65–67]	In your free time on an average week, how many hours do you spend doing the following activities? [0 h, ½ h, 1 h, 2 h, 3 h, 4 h, 5+ h] • Watching TV/DVDs/videos • Using a computer • Electronic games that you play when sitting	Select the activity that most closely matches what you are doing (not including responding to this assessment). • Reading/attending class/doing homework/studying • Hanging out • Texting/using phone • Watching TV or movie • Browsing the internet • Using social media on the internet • Playing video games • Sleeping/lying in bed • Working/attending a meeting • Showering/getting ready • Eating • Other (specify): In total, how much time will you spend on this activity? [<30 min, 30 min–1 h, 1–2 h, >2 h]	--
Weight status	• Measured height, weight, waist and hip circumference • Self-reported height and weight	--	--
Predictors and moderators/mediators			
Friendship network and friends	Identify your 5 closest male friends and 5 closest female friends in your residence hall [34–37] • List them in order of closeness (best friend first) • Identify your roommate	Who is with you? • I am by myself • Friend(s) ○ Select which of the following friends were with you (a list of the participants nominated friends will appear. Participants can choose none of the above) ○ Other friend(s): ○ How many female friends? ○ How many male friends? • Roommate • Classmates/peers/coworkers • Family • Significant other (select) • Other (specify):___________	Time stamp and location; friends’ data will be linked
Friendship-network mechanisms	• Time spent with friend(s) on eating/PA/sedentary activities • Type of friends • Length of friendship • Friendship closeness [38] • Friendship maintenance [39] • Openness to friendship [40–42]	Select if any factors below were involved in your [eating/drinking/PA/inactive PA] (Please check all that apply) [8, 68]. • Scheduled time (for meals, gym, work out class etc.) • Friend(s) suggested it • Food/Drink/Gym or opportunity was easily available • In lecture/lab or similar • Others do it, so I feel I should too • Wanted to celebrate • I saw someone else doing it • None of the above • Other (specify): _____ If you were alone, would you have made the same choice in eating/drinking/being active/not being active?	--
Geospatial place	--	Automatic GIS longitude/latitude coding embedded into EMA app	Location of expenditure
Time	--	Automatic time stamp embedded into EMA app	Automatic time stamp

### Participants

We aim to saturate the residence halls (i.e., recruit all freshmen) to have as complete a friendship network as possible (close friends, roommates, friend groups, and networks at the residence hall floor and residence hall level will also be included). Residence Life and Resident Assistants (Community Mentors and Community Assistants) from each residence hall helped to facilitate recruitment.

### Incentives

For each completed assessment, participants earn incremental monetary awards (up to $110) and additional earned incentives (“swag”: e.g., study branded water bottles, t-shirts, Frisbees, ear buds, tote bags). In order to not impact the social network of the study, floor-level incentives (e.g., pizza party) are not offered. When floors reach 60 % or higher enrollment, individual participants are offered additional swag of their choice. Community Mentors and Assistants are offered $15 gift cards if their floor reaches 60 % participation and $40 in gift cards if their floor reaches 80 % participation at each data collection point.

### Measures

Web-based surveys and anthropometric measurements: The web-based surveys address personal, interpersonal, and environmental factors related to participants’ weight and weight related behaviors and takes 20–30 min to complete. Included in the survey are validated measures of eating, PA, and weight status, as well as a series of questions about participants’ relationships with friends. Baseline demographic characteristics (e.g., age, gender, race/ethnicity, Pell Grant status, and parental education) are collected at the first data collection point.

The validated 26-item Dietary Screener Questionnaire (DSQ) used in the 2010 National Health Interview Survey Cancer Control Supplement, a free tool developed by the National Cancer Institute of NIH [25, 26] assesses the frequency of consumption of key food items and groups. While this tool does not estimate individual’s caloric intake, it allows for tracking of the consumption of major food groups (e.g., fruits and vegetables, high fat foods, sugar sweetened beverages) related to weight. In addition, participants are asked to report how often they ate breakfast [27], evening meals [28], and fast food [27]. The USDA six-item food security short form is included to examine changes in food security status [29].

PA is examined with the Godin-Shepard PA assessment [30, 31], which assesses usual vigorous, moderate, and light PA: “In a usual week, how many hours do you spend doing the following activities: Strenuous exercise (heart beats rapidly)?; Moderate exercise (not exhausting)?; Mild exercise (little effort)?” Response options ranged from none to more than 6 h per week. A sum of the time spent in PA and moderate-to-vigorous PA will be created. Sedentary activities are assessed with the question: “Yesterday, how much time did you spend in front of a screen (excluding time in class and being physically active)?” Response options ranged from zero to more than 6 h and will be summed to create a total time spend in sedentary behaviors [32, 33].

Participants complete friendship network questions on each web-based survey for which they list their 5 closest male and female friends [34–37] and report the time spent with them eating, being physically active, or sedentary. Participants also respond to how long they have been friends with that person and whether or not the friend is their best friend, roommate, suitemate, or significant other. Participants are asked to indicate their level of closeness with each nominated friend [38] and how they maintain their friendship with each person [39]. In addition, participants are asked to reflect on their openness to new friends while on campus [40–42]. These survey data will be linked among nominated friends and roommates to assess associations over time with questions about weight-related behaviors and outcomes at each of the four time points.

Height and weight are measured privately by trained research staff at the same times as the web-based survey using Seca scales and stadiometers to track changes in body mass index. Waist and hip circumference are collected using flexible tape measurers. Participants are asked to self-report on their height and weight, as well as whether they are trying to change their weight in any way [43].

mEMAs: During each wave of mEMA data collection, students are prompted via SMS text messages to complete the mEMA eight times per day on four of the 7 days in their assessment period, with at least 1 weekend day per wave. As such, a potential for 128 repeated measures for each participant is possible with the mEMA. A random, interval-contingent schedule is used for the mEMA prompts. Twice during each of the four established time periods per day (9 am–12 pm, 12–3 pm, 3–7 pm, and 7–10 pm) the system randomly prompts participants to complete a brief survey. In order to ensure the momentary nature of the mEMA, participants are allotted 35 min to respond to the prompt by completing a 1-min survey, with the survey being available for 5 min prior to, and 30 min after, the text message prompt. Outside of these times, the mEMA surveys are not available to complete on the app. Because of Apple restrictions, the devilSPARC app was developed through Apple Enterprise and is available only through a study website. Trained research assistants assist with downloading the devilSPARC mEMA app to each participant’s smartphone and provide demonstrations at each data collection period on how to use the devilSPARC app. For participants who do not have android or iOS operating systems on their smart phones (*n* = 27), a data-enabled smart phone is loaned to them for the 1 week duration of each wave of mEMA data collection, a method that previous studies found successful [44, 45]. If participants have a new phone or need to update the app, trained research assistants assist with downloading the devilSPARC mEMA app. In addition, each participant receives paper instructions on how to download the app and detailed instructions are emailed and posted on the study website.

**Fig. 2:**
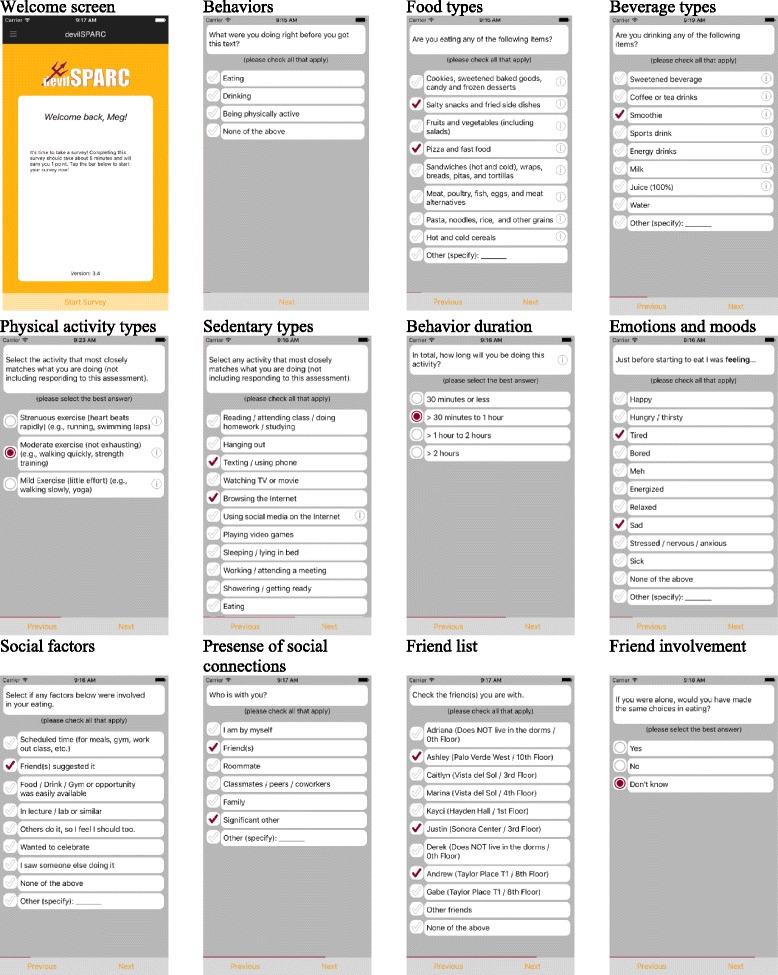
Participant view of the mEMAs from an iOS system

To minimize participant burden and increase response rates, each mEMA is limited to 5–8 questions as has been suggested in previous research [46, 47] addressing different weight behaviors and contextual factors about the behavior (e.g., who the participant is with and how they are feeling). Depending on the behaviors that the participant reports, a skip pattern is enabled (e.g., if participants are not being physically active, then questions about their physical activity are not viewable). Eating behavior measures in the mEMA are based on common foods related to weight among college students as identified by Laska and colleagues [48] and also foods reported in 24 h recalls in validation testing of the app [49]. See Fig. 2 and Table 2 for detailed descriptions of the mEMA items.

Student card data: Students use their student card to purchase food, attend university activities, and access facilities such as dining halls and recreation centers. University databases will provide student card data for students’ purchases and activities related to eating and PA, which will be linked to their friendship networks’ activities. The mEMA data will also be matched with participants’ student card activity. With this information (e.g., student access to campus recreation centers, purchases at certain food outlets and the dining halls), we will capture additional behavioral, temporal (time stamp), and geospatial data on what friends are doing together.

### Statistical analyses

To analyze the collected data, we will use mixed model regression techniques to develop and test egocentric models (analysis derived from an “index” participant) over the course of the year [50]. Egocentric models will include associations between index student behaviors and behavioral measures of corresponding friendship networks (residence hall-level and floor-level) and friends (e.g., roommate, close friends and friend group). We will also examine moderation of these effects by contextual factors, such as location, time of day, and individual differences (e.g., gender, race/ethnicity). Friend effects require special attention in egocentric models. If two individuals in the study are friends with each other, then each person’s data could be used as both an outcome and a predictor in the same analysis, thus violating the generalized linear model’s assumption of independence among observations. Accordingly, we will use a generalized linear mixed (or multilevel) model framework. Due to the sampling design of this study, empirical clustering among students’ responses within residence halls and within floors of residence halls is likely. If warranted, we will account for these additional sources of non-independence among observations by including random intercept effects for different levels of nesting (e.g., index participants within floors, floors within residence halls). Effects of age, gender, race/ethnicity, and socioeconomic status (SES) will be adjusted for as necessary. Assumptions regarding linearity of associations and homoscedasticity will be checked via analysis and visual inspection of residuals. Further, we will test for temporal autocorrelation in the data and, as necessary, account for it (e.g., by specifying autoregressive error structures in our models). The choice of link function (e.g., identity, logit) and error distributions (e.g., Gaussian, binomial, Poisson) will correspond to the nature of the outcome measure being modeled. Though some continuous outcomes may not be normally distributed, based on the central limit theorem, we expect the large sample size proposed here to ensure that model estimates will be asymptotically unbiased [51]. Conservative power analyses indicate that the projected sample size of 1100 cases should afford power of .80 to detect small effects (ds < .25), assuming residence hall-level ICCs of .01 and alpha of .05.

Stochastic actor-oriented models (SAOMs) [52, 53] will be used to analyze influence on key outcomes while controlling for selection into friendships. The SAOM is a longitudinal model with separate functions to estimate change in behavior and change in friendship networks due to varied selection processes. This model form allows both behavior and friend selection to be modeled endogenously, such that we can untangle issues related to homophily (i.e., students choosing similar friends) and contagion (i.e., transmission of ideas from friend to friend) [21]. The SAOMs will also distinguish between the influence of friends, best friends, and roommates as predictors of individual behavior. The SAOMs will account for the embedded clustering in the sampling design due to students being sampled from floors within residence halls, constituting a multilevel network (i.e., floor networks nested within residence halls). SAOMs require “complete” network data that includes most individuals within a bounded setting, hence the saturated sampling design. Social network analyses is possible with the high saturation rates. For this study only floors with response rates in the neighborhood of 75 % saturation or greater will be included in the SAOM analysis. SAOMs will be used in addition to the egocentric models to address the study aims.

## Results of the pilot studies

Since the study is ongoing, we will present our results to date. In 2014–2015, we conducted pilot studies of our protocols. In total, 304 college freshmen and their Community Mentors (mean age = 18.9 + 0.60; 62 % female; 52 % non-white) participated. In three pilot tests, we conducted test-retests [54] and validated the eating and PA measures in the mEMA against 24-h dietary recalls and accelerometry, respectively [49]. Among participants (*n* = 109) who were asked to test the usability and functionality of the mEMA, there was a 66 % compliance rate (range: 6–100 %; median = 72 %).

In these pilot studies, we also examined different approaches to recruitment. In two residence halls, we recruited from residence hall lobbies and court yards. In one residence hall, we recruited from mandatory floor meetings (we invited residents to participate in the study at the end of meetings that reminded residents of policies and procedures for living in the residence hall). In the residence halls where we recruited as students were coming and going, we were able to achieve a 42 % saturation rate over the span of 8 days and over 45 h of data collection. In the residence hall where we recruited from the floor meetings, we achieved a 45 % saturation rate over the course of 3 days and approximately 20 h of data collection. We found the most effective recruitment strategy was to have genuine encouragement from the Community Mentors. We also invited the Community Mentors to participate in the study in the residence hall where we collected data from the floor meetings, and observed higher enthusiasm for the study among participants in that residence hall.

## Discussion

The current study is following a diverse body of college freshmen through a full academic year, allowing better identification of (1) the mechanisms by which friends have an impact, (2) how persistence and strength of relational ties affect health behaviors over time, and (3) the contextual factors that modify these relationships. With these data integrated at multiple time points, we are able to examine how friendship networks, friend groups, and/or close friends/roommates are doing similar activities over time. By assessing the temporality of the relationships through intensive real-time repeated measures, we are able to assess how different mechanisms (such as norms and selection) impact participant behaviors and begin to address the causality of the associations between friends and behaviors.

We launched the full study in August of 2015 with four residence halls from one academic residential college on one campus. A total of 1557 students were eligible for participation from these four residence halls. A team of over 50 undergraduate and graduate students, staff, and faculty visited each floor (*n* = 30 floors) in teams of 15–20 of the four residential halls the day after the students arrived on campus. We returned 2 days later for additional recruitment and then also recruited from the lobbies of each of the buildings in the following week. By September, 716 students had enrolled in the study. Given that the saturation of these residence halls was less than ideal (~46 % dorm level saturation; 40 % floor level saturation), we expanded our reach from four residence halls on one campus to a total of six residence halls on three campuses (within the same university located in the same metropolitan area). We enhanced incentives to promote the inclusion of friends by offering a refer-a-friend bonus, where existing SPARC participants received an additional $5 gift card each time an eligible friend enrolled in the study. Participants who refer a friend to enroll in the study could also receive an entry into a raffle for a chance at $100, $50, $25, and $10 bonuses. To encourage participation across waves, we instituted additional incentives at each time point: $20 cash raffles are available at each day of data collection in each residence hall; if participants complete at least 75 % of the mEMAs in a given wave, they can earn an additional $5 gift card; at the last wave of mEMAs, participants can be provided an extra $5 bonus for the first five surveys completed. To enhance our saturation, we continue to enroll new participants through web-based survey #3, which would allow for at least two in person data points, with two mEMA waves.

As an emerging area of social epidemiology, the role of friendship networks in weight-related behaviors and outcomes has other key inconsistencies and gaps [10, 11, 55–58], some of which we have also attempted to address in our design. Most of the existing data are cross-sectional [14, 59, 60], and those that have included longitudinal measures have included limited time points and relatively small sample sizes [11, 56, 61, 62]. Because of the methodological limitations in previous studies, the mechanisms by which friends impact eating and PA behaviors and weight outcomes are not clear [21, 23, 24]. The current study purposefully triangulates quantitative data; each method will provide information on behavior and friends, allowing for corroboration of findings from the complementary data collection efforts [24]. The rich dataset will include multiple layers, including individual and interpersonal behaviors/outcomes and environmental factors; the mEMAs provide a means to better integrate the diverse sets of behavioral and attitudinal data through systematic temporal and proximity tracking of participants.

Overall, our pilot data confirm indicated acceptability and feasibility of the SPARC study protocols. We found that recruitment was most effective and efficient when paired with an existing floor meeting. Community Mentors’ (residence assistants’) excitement and engagement with the study was related to higher participation rates on respective floors. With competing interests and staffing constraints, our longitudinal enrollment was less than anticipated. For example, all freshmen were invited to participate in a university-wide app study on health behaviors at approximately the same time that our study started. As such potential participants were confused about the differences between the studies and to which study they would like to enroll. While the total sample size was within our target, we had to enroll students from additional campuses. Our average floor saturation was approximately 40 %, which means we will rely primarily on ego-centric analyses, with the more sophisticated SAOM analysis reserved for floors with more complete saturation. An advantage of the ego-centric approach in the current study is that we have data from those friends who participated in the study; such self-reported data are less likely to exhibit projection bias than the simpler method of gathering proxy reports of friends by respondents.

To our knowledge, no studies have used EMAs or mEMAs to assess friendship networks and health behaviors and outcomes [63]. Using mobile-based technology to collect EMA data is also innovative and will likely result in higher participation rates than other EMA paper-and-pencil approaches, especially among a population that has the highest use of smart phones [64]. Information gleaned from working with college students will help in designing feasible and acceptable EMA observational and intervention studies for other populations. Our pilot data suggests that mEMA was relatively acceptable among participants. The technology worked well and we collected all of the behaviors, social context, and geographic data that we anticipated we would. However, in the longitudinal study, we have experienced some challenges with the mEMA. In particular, Apple released a new operating system between waves one and two of the mEMA. For students who updated their operating system to iOS9, the app would crash when opened. Unfortunately, over 80 % of our participants had an iOS phone, but we did not know which participants updated their operating system. As such, we offered a $5 bonus for downloading a new version of the app that would not crash.

Given the challenge of unhealthy eating habits and PA behaviors and rates of obesity among college freshmen, innovative research will help scientists better understand the contributory factors. Relatively few studies have examined the role of social factors on these problems, and here we have provided an example of the types of technology-driven tools that can open this as a valid and reliable field of enquiry. Despite encountered challenges, given the longitudinal nature and intensive data collected, we will still be able to answer the study’s primary research questions: to describe the mechanism(s) by which friends’ and freshmen’s eating/PA behaviors and weight are related and to examine contextual factors related to behaviors among friends over time. The information gleaned from this study will be used to test and develop obesity prevention interventions among friends and social networks.
